# HIV prevention research and COVID-19: putting ethics guidance to the test

**DOI:** 10.1186/s12910-021-00575-w

**Published:** 2021-01-25

**Authors:** Stuart Rennie, Wairimu Chege, Leah A. Schrumpf, Florencia Luna, Robert Klitzman, Ernest Moseki, Brandon Brown, Steven Wakefield, Jeremy Sugarman

**Affiliations:** 1 UNC Center for Bioethics, University of North Carolina at Chapel Hill, Chapel Hill, NC USA; 2 Division of AIDS, National Institute of Allergy and Infectious Diseases, National Institutes of Health, Rockville, MD USA; 3 Family Health International, Durham, NC USA; 4 FLACSO-Argentina and CONICET, Buenos Aires, Argentina; 5 Columbia University, New York, NY USA; 6 Botswana Harvard AIDS Institute Partnership Princess Marina Hospital, Gaborone, Botswana; 7 Center for Healthy Communities, University of California Riverside School of Medicine, Riverside, CA USA; 8 NIAID HIV Vaccine Trials Network, Seattle, WA USA; 9 Berman Institute of Bioethics, Johns Hopkins University, 1809 Ashland Ave, Baltimore, MD 21205 USA

**Keywords:** HIV prevention, Research ethics, COVID-19

## Abstract

**Background:**

Critical public health measures implemented to mitigate the spread of the novel coronavirus disease (COVID-19) pandemic have disrupted health research worldwide, including HIV prevention research. While general guidance has been issued for the responsible conduct of research in these challenging circumstances, the contours of the dueling COVID-19 and HIV/AIDS pandemics raise some critical ethical issues for HIV prevention research. In this paper, we use the recently updated HIV Prevention Trials Network (HPTN) Ethics Guidance Document (EGD) to situate and analyze key ethical challenges related to the conduct of HIV prevention research during the COVID-19 pandemic as well as identify potential areas for refinement of the guidance document based on this unprecedented state of affairs.

**Main body:**

Necessary actions taken for HIV prevention research studies due to the COVID-19 pandemic involve an array of ethical issues including those related to: (1) risk mitigation; (2) behavior change; (3) compounding vulnerability; (4) community engagement; (5) trial reopening; and 6) shifting research priorities.

**Conclusions:**

In the context of the dueling HIV and COVID-19 global pandemics, research teams and sponsors must be nimble in responding to the rapidly changing environment by being sensitive to the associated ethical issues. The HTPN EGD provides a rich set of tools to help identify, analyze and address many of these issues. At the same time, future refinements of the HPTN EGD and other research ethics guidance could be strengthened by providing explicit advice regarding the ethical issues associated with disrupted research and the reopening of studies. In addition, additional consideration should be given to appropriately balancing domains of risk (e.g., physical versus social), addressing the vulnerability of research staff and community partners, and responding to un-anticipatable ancillary care needs of participants and communities. Appropriately addressing these issues will necessitate conceptual work, which would benefit from the careful documentation of the actual ethical issues encountered in research, the strategies implemented to overcome them, and their success in doing so. Throughout all of these efforts, it is critical to remember that the HIV pandemic not be forgotten in the rush to deal with the COVID-19 pandemic.

## Background

Critical public health measures implemented to mitigate the spread of the novel coronavirus disease (COVID-19) pandemic pose substantial challenges for efforts to curtail the ongoing HIV/AIDS pandemic. In attempt to attenuate the COVID-19 pandemic, governments and health authorities have worked to “flatten the curve” of incidence through a variety of initiatives. Some involve changing behaviors; for example, frequent handwashing, wearing face coverings, maintaining physical distance, enacting orders to “shelter-in-place”, and requiring self-isolation or quarantine as appropriate [1]. Others involve structural changes: shutdowns of businesses, limits on numbers of people allowed in establishments, and limits to transport usage. Unfortunately, such measures also make it difficult or impossible for many people to access essential medical care and services, including HIV care and prevention services [2, 3]. Similarly, much health research has been disrupted. While guidance regarding these disruptions has been issued for this research in general [4–8], the contours of the dueling COVID-19 and HIV/AIDS pandemics raise some critical ethical issues for HIV prevention research, not only in the short-term for current research, but also in the long-term in the event of subsequent waves of COVID-19 or other pandemics.

The HIV Prevention Trials Network (HPTN) is a global research network that develops and tests the safety and efficacy of interventions in multi-site research projects designed to prevent the transmission of HIV. The authors of this article are members of the HPTN Ethics Working Group (EWG), which is charged with providing ethics expertise and guidance to inform the design and implementation of HPTN research [9]. The EWG includes experts in the field of ethics of human subjects’ research, members of the HPTN leadership and Operations Center, and the Division of AIDS (DAIDS) of the National Institute of Allergy and Infectious Diseases at the US National Institutes of Health [10, 11]. More details on the composition of the EWG can be found on its website [9]. The HPTN EWG is also responsible for updating the HPTN ethics guidance document (EGD), which is integrated within HPTN research protocols and procedures. The HPTN EGD was initially created in 2003 and the current third edition was issued in February 2020 [10, 11]. The EGD is considered a leading normative framework in HIV prevention research ethics [12, 13]. The EGD covers the entirety of the research continuum, from study design and objectives to post-trial obligations, offering fifteen interrelated ‘ethics guidance points’ (See Table 1).

**Table 1:** HPTN Ethics Guidance Points

Number	Summary
1	Those engaged in HIV prevention research must be committed to designing and implementing high-quality scientific research and research ethics practices throughout the research process
2	HIV prevention research should prioritize efforts that address public health needs, reduce health inequities, and are locally relevant
3	Relevant communities should be actively engaged throughout the research process to help ensure that HIV prevention research is appropriate as well as scientifically and ethically sound
4	HIV prevention research should seek to develop local capacity and establish collaborative partnerships
5	HIV prevention research should be designed to minimize risks and maximize benefits to study participants and their communities, while remaining scientifically sound
6	Each site involved in HIV prevention research should develop, implement, and document appropriate informed consent, assent, permission, and re-consent processes tailored to the needs of participants
7	HIV prevention researchers should assess, monitor, and respond to the social, cultural and other factors that may place research participants at heightened risk
8	Independent ethics review committees in host countries should review HIV prevention research
9	HIV prevention researchers should partner with key stakeholders to provide a package of effective, comprehensive, and sustainable prevention services to all participants in HIV prevention research
10	HIV prevention researchers should strive to provide care and treatment to participants that exceed local standards of medical services yet does not impose undue influence to participate in research
11	HIV prevention researchers and sponsors should ensure that appropriate mechanisms for independent data and safety monitoring are in place
12	HIV prevention researchers should plan for the timely communication of HIV prevention research results to scientific audience as well as participants, affected communities, and other stakeholders in a manner that promotes understanding and trust
13	HIV prevention researchers should endeavor to ensure that the investments made in developing capacity will continue to provide benefits and opportunities for local researchers and communities after research ends
14	HIV prevention researchers should seek to facilitate continuity of prevention services and care for participants who still require it after research participation has ended
15	HIV prevention research seeking to establish the efficacy of an intervention must have at minimum a preliminary plan regarding post-trial access to interventions proven to be safe and effective, which offer meaningful benefit for research participants and their communities

In this article, we use the latest version of the HPTN guidance to situate and analyze key ethical challenges related to the conduct of HIV prevention research during the COVID-19 pandemic as well as identify potential areas for refinement of the EGD based on what has been encountered during this unprecedented state of affairs.

## Main body

As the COVID-19 pandemic emerged, the HPTN immediately implemented several measures to protect the safety of participants and staff: no new studies were initiated; observational studies were paused; screening and enrollments in ongoing non-observational studies were paused; and follow-up continued for participants currently enrolled in specific HPTN studies, with guidance provided from DAIDS and the HPTN regarding optimal ways to do so [14]. In addition, there are continuous modifications of ongoing interventional studies, taking into account changing local environments, including local policies, staff safety and site capabilities.

Nevertheless, these necessary actions and the circumstances due to the COVID-19 pandemic involve both long and short-term ethical implications for HIV prevention research related to: (1) risk mitigation; (2) behavior change; (3) compounding vulnerability; (4) community engagement; (5) trial reopening; and (6) shifting research priorities. We examine these in turn (Table 2).

**Table 2:** Ethical Issues Encountered in HIV Prevention Research due to the COVID-19 Pandemic and Corresponding HPTN Guidance Points (GP)

Action/Circumstance	Issues	GP
Risk Mitigation	Physical safety of study participants, research personnel, and community partners Economic and social risks for study participants	5
Behavior Change	Changes in risk behaviors influencing event rates Changes in use of PrEP and other means of prevention	1
Compounding Vulnerability	COVID-19 pandemic disproportionately effects populations many of which are similarly effected by HIV/AIDS Adverse physical, psychosocial and economic effects Responding to COVID-19 at the expense of other health concerns	7
Community Engagement	Need to maintain community engagement during study interruptions and pauses Challenges to community engagement	3
Trial Re-opening	Potential risk of increasing COVID-19 transmission Preserving scientific validity and social value of HIV prevention research	1, 5
Shifting Research Priorities	Neglecting research on other diseases Delay in identifying new effective strategies for reducing HIV acquisition in at-risk populations	2

### Risk mitigation

In the HPTN EGD, Guidance Point (GP) 5 states that HIV prevention research should be designed to minimize risks and maximize benefits to study participants and their communities, while remaining scientifically sound. While the EGD does not directly engage with issues related to the conduct HIV prevention research during massive public health crises, it does anticipate the integration of electronic tools and platforms into study operations, the use of which have now become imperative to help decrease SARS-CoV-2 transmission risk in HIV prevention research. The EGD also acknowledges that these useful tools and platforms carry their own risks, such as privacy concerns with wearable devices and online interactions, the potential for participant fraud and misidentification, and the risks of inadvertent disclosure of HIV status, study participation, and stigmatized behaviors. Of course, such concerns may also pertain to ‘old school’ methods such as receiving biological samples or sending HIV self-testing kits by mail and conducting safety evaluation visits by phone, yet they may be amplified or arise somewhat differently with the use of new technologies [15]. Regardless, the scientific integrity of many ongoing HIV prevention studies would be impossible or seriously compromised if every study interaction conducted remotely; and conducting high-quality research is non-negotiable (GP 1, more below). For example, in the forthcoming HPTN 094 INTEGRA study, the use of a mobile clinic to deliver ‘one-stop’ integrated health services to people who inject drugs will be tested in five U.S. cities. This intervention cannot be implemented virtually. Following GP 5, in-person research interactions are ethically permissible if and only if they could not be conducted remotely, are necessary to the study objectives, and when protocols are in place to minimize the number of visits and amount of exposure to participants and study staff. Accordingly, private transport was arranged for participants in the Brazil site of HPTN 083 (NCT02720094, which is testing a long acting injectable form of pre-exposure prophylaxis) and HPTN 085 (NCT02716675, which is testing the safety and efficacy of a human monoclonal antibody for HIV prevention) rather than have participants run transmission risks on public transport. In contrast, following directives of the Vietnamese Ministry of Health, the Vietnam site for HPTN 083 was closed for three weeks after a COVID-19 outbreak at Bah Mai Hospital. During that period, no in-person study visits were conducted, but communications with participants continued via phone or social media, which provided updates about the site closure, scheduled visits for after site reopening, and answered questions and concerns. At the Malawi site that was participating in HPTN 084 (NCT03164564, a study also evaluating a long acting injectable form of pre-exposure prophylaxis), structural changes such as screening at entry to buildings and referral of suspected COVID-19 cases to clinics external to the research site were implemented. At the Malawi and Vietnam sites, research-related COVID-19 risks were also mitigated by external factors, such as relatively low COVID-19 incidence and early implementation of stringent public health restrictions, which helped make it possible for in-person research visits to continue.

Like the Belmont Report [16] the HPTN EGD understands risk broadly to include physical, psychological, legal, social and economic risks, which should all be minimized to the extent possible and balanced with potential benefits. However, in the context of the COVID-19 pandemic, doing so is especially challenging. For example, altering research procedures to minimize physical risk may increase social risks due to the loss of interaction between participants and study staff, adding to the mental and emotional toll of increased physical distancing and sheltering in place. Balancing physical and social risks will be study- and population-dependent, as not all participants will value these interactions in the same way or be similarly willing to risk in-person visits. In addition, loss of employment and resources due to COVID-19 may motivate participants to risk COVID-19 exposure in order to continue participating in HIV research that offers healthcare, incentives, or other tangible benefits unlinked to the research. The EGD had not explicitly considered such tradeoffs among different categories of risk. Nonetheless, as a result of these tensions, decisions concerning particular research activities should include participant perspectives where feasible [17], following GP 3 (below). Nevertheless, accommodating some participant views and preferences (such as unwillingness to wear masks or maintain social distancing) would be ethically unjustified. Furthermore, while researchers should be aware of these emerging motivations and views, and be sensitive to them, protocols should be developed to reduce research-related COVID-19 exposure risks.

### Behavior change

According to GP 1, those engaged in HIV prevention research must be committed to designing and implementing high-quality scientific research and research ethics practices throughout the research process. The COVID-19 pandemic has complicated the fulfillment of these fundamental obligations in a variety of ways including limiting the ability to provide study interventions and assessing outcomes as described above as well as by changing the sexual and other behaviors of study populations involved in HIV prevention trials. For instance, the pandemic may make illegal drugs more expensive and difficult to obtain, leading individuals to switch drugs [18], which in turn may change the HIV risks faced by participants or the relevance of particular interventions in this context.

In addition, the media have suggested that many men who have sex with men (MSM) are not using oral pre-exposure prophylaxis (PrEP) because they are not having sex, while others deal with stress and isolation by having sex [19]. Rumors have also circulated that PrEP may help prevent COVID-19 [20]. More generally, in a study of U.S. MSM, COVID-19 was found to have a wide range of socioeconomic and behavioral impacts. Overall, respondents had fewer sex partners (51.3%), fewer opportunities to have sex (68.0%), and less use of condoms (9.4%). About 10% used more recreational drugs, and 26.0% used more alcohol. Respondents also reported decreased access to HIV testing (18.8%), HIV care visits (27.0%), viral load or other lab testing (23.8%), sexually transmitted infection (STI) testing or treatment, and antiretroviral (ART) prescriptions (8.2%). As such, it may be difficult to properly understand HIV risk and to assess the effects of preventive interventions. Such profound and sudden changes also raise questions about the extent to which research findings gathered during COVID-19 pandemic are generalizable to post-COVID-19 circumstances [21]. Thus, the COVID-19 pandemic can challenge the social value of some HIV-related research.

Regardless, acting in accordance with the HPTN EGD may necessitate making relevant changes to study procedures and/or gathering knowledge of behaviors during the pandemic yet doing so poses practical and ethical challenges. For example, for HIV prevention studies involving oral PrEP (whether as an experimental arm or as standard of prevention), investigators may need to closely monitor adherence to PrEP or other HIV prevention interventions during the COVID-19 pandemic, and not simply assume that participants have continued to use them for any number of reasons (e.g., availability, changed sexual behaviors). Some remote monitoring methods have been proposed, such as providing SMS photographs of pill counts and refill dates, and mailing in samples of hair that can be used to measure medication concentrations [22]. Researchers should also communicate with participants about whether to continue PrEP if they are not sexually active due to social distancing. Zoom or other video chat platforms could facilitate such interactions, at least for participants with reliable internet access. In addition, researchers should reasonably assume that some study participants will not follow social distancing guidelines and engage in sexual activities; participants who engage in survival sex work during the COVID-19 pandemic are at particular risk for infection, violence and psychological harm [23]. More generally, since the COVID-19 pandemic is psychologically challenging, appropriate counseling for individuals at risk for HIV is critical to enable them to make informed decisions about PrEP and risk behaviors. But while monitoring, communicating and counseling can help to counteract the impacts of COVID-19 on study integrity as described above, they also increase risks of inadvertent HIV status disclosure, loss of confidentiality and stigma. Finding the right balance between these considerations will depend on specific contextual details. Inputs from social science, community engagement activities and research ethics communities could help identify and mitigate potential social harms raised by attempts to adjust study procedures to behavioral changes driven by COVID-19.

At the extreme, COVID-19 related behavioral changes may be so profound, and the capacity to conduct new research on behavior during the pandemic is so hampered, that a study becomes irretrievably compromised. Behavior change to this extent can be a reason for study termination (see Trial Reopening, below). Given the social value of the research as initially proposed and implemented, a decision regarding termination would need to be predicated on a convincing demonstration of how the study as designed or modified cannot reasonably meet standards of scientific validity. Even in such a dire situation, researchers still have an obligation to salvage whatever remains of value (e.g., study instruments, collected data), even if a far cry from the original study objectives.

### Compounding vulnerability

GP 7 considers vulnerability in terms of factors or layers that place the health and well-being of individuals at heightened risk in their daily lives and particularly states that HIV prevention researchers should assess, monitor and respond to the social, cultural and other factors that may place research participants at heightened risk. The corresponding obligation on the part of researchers is to avoid exploiting or exacerbating existing factors for vulnerability and to minimize them when feasible and appropriate. The COVID-19 pandemic is compounding vulnerabilities by disproportionately affecting those already subject to poverty, discrimination, poor health, food insecurity, gender based violence, and stigma such as African-Americans, Latinos, older persons, and undocumented immigrants. In many ways, this parallels the disproportionate effect of HIV on racial, sexual and ethnic minorities. In short, persons at heightened risk for acquiring HIV—the population of interest for HIV prevention research—were already vulnerable in many different and intersecting ways, and now they must also contend with the adverse impacts of COVID-19.

In their efforts to minimize the potential to compound vulnerability, researchers should collaborate with community partners to identify key vulnerability factors in the community where HIV prevention research is being conducted. Besides promoting better study procedures generally, this could help raise awareness that procedures designed to reduce risk of COVID-19 transmission in HIV prevention research may have the unintended effect of increasing participant vulnerability. For example, the vulnerability of participants who were comfortable with in-person clinic visits may be increased if contacted via online platforms by study staff, depending on their domestic situations; homeless persons may find themselves alienated or excluded by protocols heavily incorporating electronic platforms; newly unemployed parents whose children are taking classes from home may be further burdened by lengthy remote research interventions; mail sent to participants containing much needed study reimbursements may be stolen if they are living in shelters or congregate settings [24]. Only through substantive engagement with the community can researchers know whether and to what extent such scenarios are likely to minimize or avoid compounding vulnerabilities.

Community partnerships may also be helpful in developing ways of mitigating study-related vulnerabilities. Local community organizations (such as those distributing food and masks) whose operations are ongoing may be more continuously in contact with study participants than research teams. These could act as a risk-reducing channels for information, particularly if held outdoors and following COVID-19 precautions. Although labor and time intensive, problems raised by the move to digital technology platforms could be eased to some extent by capacity-building and technical assistance efforts on the part of research teams.

In addition, as some have pointed out, the vulnerabilities of community partners (including health care workers) involved in HIV prevention research are also being exacerbated by the COVID-19 pandemic, manifesting in heightened exposure risk, burnout, moral distress and economic insecurity [25]. Although not a focus in the HPTN EGD, providing support for those in the community who facilitate the conduct of research in a time of crisis is ethically justified on the basis of solidarity and reciprocity. What form this support takes (such as sharing medical equipment or providing some mental health services) depends on site resources, funding constraints and input from community stakeholders.

### Community engagement

As GP 3 emphasizes, relevant communities should be actively engaged throughout the research process to help ensure the HIV prevention research is appropriate as well as scientifically and ethically sound. Community engagement is an interactive relationship between researchers and stakeholders including study participants and communities from which they come [26] and is based on mutual respect, autonomy and transparency [27]. Responsive lines of communication can identify and respond appropriately to rumors and misconceptions surrounding the research. As mentioned earlier, community engagement is key to the responsible conduct of HIV prevention research during the COVID-19 pandemic. However, the changes to common life brought about by the pandemic not only disrupt community engagement, but they also raise the possibility that some community engagement activities may increase risks and vulnerabilities to participants, bystanders, and researchers.

In this respect, COVID-19 has exposed a lacuna in the HPTN ethics guidance. Like similar documents, the HPTN ethics guidance primarily devotes its attention to the dangers of neglecting community engagement; that is, neglect expressing lack of respect and undermining the ability to conduct and complete important HIV prevention research. The COVID-19 pandemic has made clear that community engagement, normally regarded exclusively as intrinsically and instrumentally good, can itself raise risks of harm. From this perspective, GP 3 might be refined to read: ‘relevant communities should be actively *and safely* engaged.’

What would active and safe engagement involve? Clearly it should not involve in-person events where social distancing requirements cannot not be maintained. As community engagement often involves representatives rather than crowds, many interactions could and should be conducted online or remotely. As long as public health considerations demand, in-person community engagement activities should only be proposed if there are serious obstacles to remote communication (such as limited access to the appropriate devices or an unreliable internet infrastructure), and even then, researchers ought to explore ways of overcoming these barriers.

However, in some lower- and middle-income countries, and disadvantaged settings within high-income ones, it may be beyond the abilities of HIV prevention research teams to enable online community engagement. In such circumstances, researchers will need to work creatively with local stakeholders, agencies and community members to conduct in-person engagement activities that are culturally appropriate, effective and keep transmission risks acceptably low. In addition, in order for other researchers and communities to benefit from novel approaches to community engagement during the COVID-19 pandemic, it would be desirable for research teams to report their experiences in this regard, including cases where the risks posed by in-person engagement activities cannot be successfully mitigated, and therefore must be paused or abandoned.

The phrase ‘throughout the research process’ in GP3 is also important. Some ongoing HIV prevention research projects have scaled back their activities, others have been paused, and some that have been paused may end up being closed. Others are being planned or their launch is delayed. Community engagement should be maintained in all these cases to the extent that it is reasonably feasible to do so. Community engagement should never ‘pause.’ At a minimum, communities and study participants have a right to know what is (or is not) going on, and ideally should be involved in decision-making even if little or no data collection is taking place.

The COVID-19 pandemic has brought into question the scope of researcher responsibilities towards the communities in which they work. When communities are in crisis and people are suffering, it is fair to ask whether ethical community engagement should involve researchers doing something to address needs that are ancillary to HIV prevention research questions, such as psychological issues related to lockdowns, continued access to medical care, intimate partner violence, and food and job insecurity. The HPTN ethics guidance recommends negotiating plans for meeting ancillary care needs with local communities before studies begin (GP 10), but many ongoing research studies have been caught off-guard by the unexpected and massive rise of ancillary care needs during the COVID-19 pandemic. For example, at the HPTN 083 site in Brazil, the most vulnerable research participants were provided with a monthly basket of food and a cooking gas voucher in response to emergent COVID-19 related needs. Clearly, research teams must reassess ancillary care plans midstream and cannot meet all emerging needs. But the magnitude of considerations that can create responsibilities on the part of researchers, such as urgency of need and lack of viable alternatives for participants, may make inaction ethically untenable [28]. Research projects that are able support local initiatives to provide emergency supplies, expertise and services to communities in crisis could sustain their relevance and enhance their trustworthiness.

### Trial re-opening

Understandably, the HPTN EGD is silent on the specific question of how to responsibly reopen research studies that have been paused in a still ongoing public health emergency involving a novel, highly infectious and potentially deadly disease. However, relevant considerations can be found within the guidance. To preserve their scientific validity and social value (GP 1), most currently paused HIV prevention trials will eventually need to reopen. However, reopening paused research studies may increase risks of COVID-19 transmission to participants and research staff (GP 5). The main ethical challenge is how to minimize COVID-19 related risks when reopening studies while at the same time achieving HIV research study aims. This challenge is further complicated by uncertainties surrounding how the COVID-19 pandemic, and responses to it, will unfold.

Reopening should be a carefully designed and inclusive process. Given the diversity of site locations and trial designs, reopening plans for different sites must be responsive to relevant public health guidelines, state laws and epidemiological data [29]. Engagement of local communities when developing reopening plans is crucial for building trust and minimizing risks, although as described earlier community engagement will need to be adapted in light of current public health restrictions. To minimize risks, reopening plans should include considerations such as the re-organization of physical spaces at sites; availability of personal protective equipment to be used by research staff and provided to participants; and how to conduct recruitment and safely obtain informed consent from new participants. In general, in-person interactions between researchers and between researchers and participants should be minimized, though this will not be possible for some study interventions, such as injections or intravenous infusions, which will need to be administered as safely as possible. Customizing reopening plans at individual sites while harmonizing research practices across sites, as well as gaining research ethics committee approvals for these changes, will likely pose significant coordination challenges. HIV prevention research protocols are also likely to change to accommodate for COVID-19 risks, and these modifications will also need to be made in such a way as to maintain scientific integrity and be approved by research ethics committees. While regulatory agencies such as the Office of Human Research Protections in the United States have provided some guidance concerning reopening, research institutions have some liberty in how they manage this issue [30]. A noteworthy approach is that taken by John Hopkins University, which has issued Return to Research Guidance and established Designated Protocol Restart Committees [31].

Regardless, the resultant reopening plans should be summarized and communicated to current research participants and communities as well as in materials used for recruitment and enrollment. Reopened HIV prevention trials will involve COVID-19 related risks and burdens that participants could not have known when they initially joined. For that reason, researchers should not only communicate these new risks, but re-emphasize that their continued participation is voluntary, and that they have the right to withdraw. In some cases, re-consent may be appropriate (GP 6). As the contours of the COVID-19 pandemic changes, some trials may again be paused, necessitating reconsideration of re-opening at an appropriate time in order to best to protect participants and research staff, reduce risks, and optimize study benefits.

In some cases, studies may be terminated. Using data from ClinicalTrials.gov, 217 clinical trials were terminated due to COVID-19 related causes between December 1^st^, 2019 and December 9^th^, 2020 [32]. The reasons given for termination are diverse, including risks to participants and staff, supply chain issues, inability to conduct essential procedures (such as surgeries), recruitment challenges, local research ethics committee restrictions, and diversion of human resources. Like reopening decisions, termination decisions should be the result of a transparent and inclusive process, and should include plans for mitigation of negative impacts of termination on stakeholders.

### Shifting research priorities

GP 2 requires that HIV prevention research prioritizes efforts that address public health needs, reduce health inequities, and are locally relevant. The main concern is the potential for exploitation: if research is not responsive to local health priorities, host communities are unlikely to benefit. While HIV prevention is a global health priority, HIV prevention research can still fail to respond to local health priorities if host communities face other, more pressing health threats. For example, in some low- and middle-income countries, HIV is no longer a public health problem to the same extent as other diseases, such as malaria and tuberculosis. In the context of the COVID-19 pandemic, to what extent should HIV prevention research ‘make way’ for COVID-19 prevention and treatment research? The question is particularly pressing in certain countries (such as the United States) where the impacts of COVID-19 are far-reaching while overall HIV prevalence and incidence is relatively low.

There are reasons to believe that HIV prevention research is in no way being made redundant by the COVID-19 pandemic. First, the ethics of setting priorities in global health research is complex [33]; in this case, it is simply not clear to what extent research resources should allocated to a novel, highly infectious disease like COVID-19 rather than more longstanding ones (like HIV or TB) associated with high mortality. Currently, part of the complexity relates to the fact that COVID-19 is likely having a detrimental impact on HIV services, including prevention services, in many countries around the world. A recent modelling study on the impact of COVID-19 on HIV services in sub-Saharan Africa suggests that HIV transmission could significantly increase due to disruptions in antiretroviral treatment supply chains, condom supplies, and peer education as well as lowered access to and quality of clinical care [34]. This indicates that HIV prevention research will continue to be important, because COVID-19 may roll back hard-fought progress in HIV treatment and prevention. Efforts to strengthen HIV treatment and prevention programs, informed by HIV research, may need to be redoubled after the COVID-19 pandemic. Second, HIV is a chronic condition for which there is effective treatment, but no vaccine and no cure, and millions of persons are living with HIV or at risk of acquiring it. COVID-19 is an acute condition (though with serious lingering effects, for some) for which treatment modalities are improving quickly and global vaccine access is at least conceivable. In short, it is very likely the need for HIV prevention research will long outlive the explosion of research activity [35] currently devoted to COVID-19. Third, HIV prevention research during the pandemic remains responsive to the needs of marginalized and vulnerable at-risk populations (e.g., transgender women and adolescents) [36–38]. De-prioritizing HIV prevention research would negatively impact decades-long hopes of ending the HIV pandemic [2] and disproportionately affect those most likely to acquire HIV. They would be casualties of the ‘dueling pandemics.’

## Conclusions

In the face of the dueling HIV and COVID-19 global pandemics, research teams and sponsors must be nimble in responding to the rapidly changing environment by being sensitive to the associated ethical issues. In doing so it is essential to ensure the protection of research participants, communities and research staff, while finding ways to maintain research integrity.

The new version of the HTPN EGD provides a rich set of tools to identify, analyze and address many of the ethical issues encountered due to the COVID-19 pandemic and the measures taken to respond to it. At the same time, future refinements of the HPTN EGD and other research ethics guidance could be strengthened by providing explicit advice regarding the ethical issues associated with disrupted research, and the reopening and termination of studies. In addition, additional consideration should be given to appropriately balancing domains of risk (e.g., physical versus social), addressing the vulnerability of research staff and community partners, and responding to un-anticipatable ancillary care needs of participants and communities. Addressing these issues appropriately will necessitate conceptual work, which would benefit from the careful documentation of the actual ethical issues encountered in research, the strategies implemented to overcome them, and their success in doing so. That is, progress in refining the HPTN EGD could be greatly enhanced by incorporating descriptions of research team experiences or systematic empirical studies of challenges encountered in the conduct of non-COVID research during the pandemic, including those outside the HIV prevention research domain. In the short term, this should also be helpful to others who are undoubtedly facing similar issues. In the longer term, this should not only help with the interpretation of HIV-related research data obtained during the COVID-19 pandemic, but also with developing effective approaches that could be employed during subsequent waves of COVID-19 incidence or other future pandemics. In the future, it is possible that the complex issues raised by COVID-19 may necessitate a separate ethics guidance document, similar to how other agencies (such as the World Health Organization) produce general and topic-specific research ethics guidance. Throughout all of these efforts and developments, it is critical to remember that the HIV pandemic not be forgotten in the rush to deal with the COVID-19 pandemic.

## Data Availability

Not applicable.

## Abbreviations

COVID-19: Novel Corona Virus Disease 2019
DAIDS: Division of AIDS
EGD: Ethics Guidance Document
EWG: Ethics Working Group
GP: Guidance Point
HPTN: HIV Prevention Trials Network
MSM: Men who have Sex with Men
PrEP: Pre-Exposure Prophylaxis
